# The mediating effect of attentional impulsivity between mindfulness and problematic smartphone use

**DOI:** 10.1186/s12888-024-05708-0

**Published:** 2024-04-18

**Authors:** Minjung Kim, Goeun Seong, Min-Jeong Jeon, Young-Chul Jung, Deokjong Lee

**Affiliations:** 1 Institute of Behavioral Science in Medicine, Yonsei University College of Medicine, Seoul, Korea; 2 Department of Clinical Psychology, Yongin Severance Hospital, Yonsei University College of Medicine, Yongin, Korea; 3 Department of Psychiatry, Severance Hospital, Yonsei University College of Medicine, Seoul, Korea; 4 Institute for Innovation in Digital Healthcare, Yonsei University, Seoul, Korea; 5 Yonsei Empathy Psychiatry Clinic, Seoul, Korea

**Keywords:** Attention, Awareness, Problematic smartphone use, Mindfulness, Impulsivity

## Abstract

**Objective:**

Problematic smartphone use has been linked to lower levels of mindfulness, impaired attentional function, and higher impulsivity. This study aimed to identify the psychological mechanisms of problematic smartphone use by exploring the relationship between addictive smartphone use, mindfulness, attentional function and impulsivity.

**Methods:**

Ninety participants were evaluated with the smartphone addiction proneness scale and classified into the problematic smartphone use group (*n* = 42; 24 women; mean age: 27.6 ± 7.2 years) or normal use group (*n* = 48; 22 women; mean age: 30.1 ± 5.7 years). All participants completed self-report questionnaires evaluating their trait impulsivity and mindfulness and attention tests that assessed selective, sustained and divided attention. We compared the variables between the groups and explored the relationship between mindfulness, attentional function, impulsivity and addictive smartphone use through mediation analysis.

**Results:**

The problematic smartphone use group showed higher trait impulsivity and lower mindfulness than the normal use group. There were no significant group differences in performance on attention tests. Levels of addictive smartphone use were significantly correlated with higher levels of trait impulsivity and lower levels of mindfulness, but not with performance on attention tests. Mediation analysis showed that acting with awareness, an aspect of mindfulness, reduces the degree of addictive smartphone use through attentional impulsivity, one of the trait impulsivity.

**Conclusion:**

Acting without sufficient awareness could influence addictive smartphone use by mediating attentional impulsivity. This supports that executive control deficits, reflected in high attentional impulsivity, contribute to problematic smartphone use. Our findings imply that mindfulness-based interventions can enhance executive control over smartphone use by promoting awareness.

## Introduction

Smartphone use has spread worldwide since the advent of the iPhone in 2007, and the current penetration rate of the smartphone has reached more than half of the world’s population [1]. However, the number of people exhibiting problematic smartphone use—a maladaptive use pattern that may include withdrawal symptoms, resulting in disrupted and dysfunctional daily life [2]—has also increased over time, regardless of the country [3]. Many studies have indicated that problematic smartphone use could be conceptualized as a type of behavioural addiction [4] with negative mental effects, such as depression, anxiety and stress [5], and physical effects, such as musculoskeletal pain and sleep problems [6].

Researches are increasingly exploring the antecedent factors of problematic smartphone use to prevent and interrupt it. Mindfulness is ‘a process of regulating attention to bring about non-judgemental awareness of current experiences and to connect with one’s own experiences in a curious and receptive manner’ [7]. Mindfulness has been shown to protect against addictive behaviours, such as alcohol abuse [8], smoking [9] and workaholism [10]. As mindfulness increases, so does awareness of the sensations, thoughts and emotions associated with cravings, which reduces addictive behaviour [11]. In particular, mindfulness is a notable protective factor in behavioural addictions [12], including problematic smartphone use. Several studies exploring the relationship between mindfulness and problematic smartphone use have suggested that the higher levels of trait mindfulness tend to be associated with a lower propensity for problematic smartphone use [13, 14]. Several studies applying mindfulness-based interventions have also suggested that promoting mindfulness is helpful in improving problematic smartphone use [15, 16].

Mindfulness is a complex concept that can be defined in many ways and is made up of several facets. Several scales have been developed and used to evaluate the level of mindfulness, and Ruth Baer defined core skills of mindfulness [17] and presented the Five Fact Mindfulness Questionnaire (FFMQ), a psychometric tool to evaluate them [18]. The five facets of mindfulness are as follows: observing, describing, acting with awareness, non-judgement and non-reactivity. ‘Observing’ means noticing and paying attention to internal phenomena (i.e. bodily sensations, cognitions and emotions) and external phenomena (i.e. sounds and smells) [19]. ‘Describing’ is a mindfulness skill that involves noticing observed phenomena and briefly labelling them. ‘Acting with awareness’ means focusing on one’s current activity in the present moment with awareness and undivided attention. These skills (observing, describing and acting with awareness) must be accompanied by acceptance, which includes refraining from judgment or criticism (‘non-judgement’) and not reacting impulsively or automatically (‘non-reactivity’) [20]. These different facets of mindfulness may be associated with problematic smartphone use at different levels. For example, one previous study reported that describing, acting with awareness, and nonjudgment were significantly negatively correlated with problematic smartphone use, while other facets were not [14]. Therefore, this study seeks to explore how each facet of mindfulness is individually associated with problematic smartphone use.

Although there are many studies showing that mindfulness is negatively associated with problematic smartphone use, there has been relatively little exploration of the mediating role of other psychological factors in the relationship between mindfulness and problematic smartphone use. Many previous studies have focused on roles of affective symptoms such as depression, anxiety, and stress [21, 22]. However, because mindfulness-based interventions improve problematic smartphone use even in the populations where depression and anxiety are not prominent [15, 23], impacts of psychological factors other than affective symptoms should be considered. For instance, previous evidences suggest that the role of impulsivity should be considered in the investigations of problematic smartphone use [4]. High impulsivity reinforces addictive behaviours by making us more susceptible to immediate pleasure and insensitive to the negative consequences that follow [24]. High impulsivity appears to be antecedent to behavioural addictions, such as gambling addiction [25], Internet addiction [26] and problematic smartphone use [27, 28]. On the other hand, impulsivity has a negative correlation with major facets of mindfulness such as awareness and non-reactivity [29], and mindfulness-based interventions have been found to be helpful in improving impulsivity [30, 31]. Taken together, we speculated that low levels of mindfulness would affect problematic smartphone use, mediated by high impulsivity. Consistent with our speculation, a previous study found that mindfulness mediates the relationship between boredom proneness and problematic smartphone use, and that this mediation effect weakens as impulsivity increases [32]. Another recent study also found that self-control mediated the relationship between mindfulness and problematic smartphone use, and impulse control was included as a subdomain of self-control in that study [16].

In the relationship between mindfulness and problematic smartphone use, in addition to the role of impulsivity, we also focus to the possible role of attentional function. ‘Paying attention intentionally’ is considered one of the core elements of mindfulness [33]. Although results are conflicting depending on which subdomains of attentional function were assessed and how they were measured [34], overall prior evidences have suggested that mindfulness-based interventions can improve attentional control [35]. On the other hand, previous studies assessing attentional control through behavioural tasks have shown that subjects with problematic smartphone use had worse performance, including slower reaction times, compared to the normal use groups [36, 37]. These evidences suggest that attentional function, like impulsivity, is also likely to mediate the relationship between mindfulness and problematic smartphone use.

Although, attention and impulsivity are deeply related [38], they are not the same concept or correlated in a single direction. Attention is a complex concept that includes several subfactors [39], and can be controlled to be goal-directed or stimulus-driven [40]. Because attention, like impulsivity, is made up of multiple components, the degree to which each component is affected by impulsivity can vary [41]. Additionally, unlike trait impulsivity, attention functions can be assessed objectively through behavioural measurements rather than subjective questionnaires [42]. Therefore, we determined that attention and impulsivity are not completely dependent on each other and speculated that it would be appropriate to separately evaluate the mediating effects of attention and impulsivity in the relationship between mindfulness and problematic smartphone use.

Like mindfulness, impulsivity and attention functions can be assessed on a variety of scales, each of which is designed to reflect different facets of impulsivity and attention. The Barratt Impulsiveness Scale (BIS), a representative scale for measuring trait impulsivity, consists of subscales of attentional, motor and non-planning impulsivity [43]. By measuring attention functions through modified forms of sustained performance tests, attentional subcomponents such as selective attention, sustained attention, and divided attention can be assessed [44]. According to previous studies, the subfactors of impulsivity and attention each have different degrees of association with executive control [45, 46]. Weakening of executive control has been suggested as a major pathophysiological factor in behavioural addictions [47]. Therefore, while exploring the relationship to problematic smartphone use, this study included several subfactors of impulsivity and attention as individual variables in the analysis.

To summarized, the aim of this study was to explore the relationship between problematic smartphone use, mindfulness, impulsivity, and attention. Firstly, we compared mindfulness, impulsivity and attention functions between subjects with problematic smartphone use and those with normal usage. Levels of mindfulness and trait impulsivity were evaluated through self-report questionnaires. Attention functions, including selective, sustained and divided attention, were evaluated through the computerized behavioural tasks. We then examined whether problematic smartphone users had lower levels of mindfulness, higher trait impulsivity, and impaired attentional functions than healthy users. We also explored whether there was any correlation between addictive use of smartphones, mindfulness, trait impulsivity, and attention functions. Afterwards, we identified the mediating role of trait impulsivity and attention functions in the relationship between mindfulness and problematic smartphone use. Through this study, we aimed to provide implications for exploring the mechanisms through which mindfulness-based interventions may help improve problematic smartphone use.

## Methods

### Participants

Participants aged 19 or older were recruited from May 2020 to September 2022 through online boards. The study protocol was approved by the institutional ethics review committee of the Yongin Severance Hospital (9-2020-0029). All participants gave written informed consent before the start of the study process. We evaluated each subject’s psychiatric history through Structured Clinical Interviews for DSM-IV [48]. Because this study included factors that may be related to cognitive function, such as impulsivity and attention, we assessed the subjects’ intelligence. We measured full-scale intelligence quotients (FSIQs) through a short version of the Korean Wechsler Adult Intelligence Scale [49].

The following exclusion criteria were applied based on these assessments: (1) major psychiatric illness (e.g. major depressive disorder, schizophrenia, bipolar disorder, etc.), (2) current psychiatric medication use, (3) IQ less than 80 and (4) history of a medical/neurological disease that could affect cognitive function. For the 104 subjects (51 men and 53 women) who passed the initial exclusion criteria, we administered additional self-reporting questionnaires to assess their affective symptoms. Previous studies have shown that subjects with problematic smartphone use have a tendency to have affective symptoms such as depression and anxiety [50]. We determined that cases with significant levels of affective symptoms may have confounding effects on the clinical presentation of problematic smartphone use. Depression and anxiety were evaluated through the Beck Depression Inventory (BDI) [51] and Beck Anxiety Inventory (BAI) [52], respectively. We ultimately excluded from the analysis subjects who reported having moderate or severe affective symptoms (BDI 20 or higher, BAI 30 or higher). Ultimately, 90 subjects (44 men and 46 women) were used in the analysis of this study.

We assessed the degree of addictive smartphone use patterns using the Smartphone Addiction Proneness Scale (SAPS) [53]. The SAPS consists of 15 items on a Likert scale ranging from 1 to 4 points, with higher scores indicating more severe problematic smartphone use. This scale was standardized on 251 male and 249 female Korean young adults, and the internal consistency (Cronbach’s alpha) was 0.814 [54]. The SAPS was structured around four subdomains: disturbance of adaptive functions (5 items), withdrawal (4 items), tolerance (4 items), and virtual life orientation (2 items). The SAPS was selected for this study because it was developed and standardized for adults of similar age (20s to 30s) in the country (the Republic of Korea) where this study was conducted. The SAPS can evaluate the clinical aspects of problematic smartphone use in a multifaceted manner, and has been widely used in several related studies [28, 55]. High-risk users were those who met the following criteria: (a) total SAPS score of ≥ 44 or (b) disturbance of adaptive functions, withdrawal and tolerance subscale scores of ≥ 15, ≥13 and ≥ 13, respectively. At-risk users were those who met the following criteria: (a) total SAPS total score of 40–43 or (b) disturbance of adaptive functions subscale score of ≥ 14. We classified the participants into a problematic smartphone use group (including at-risk and high-risk groups) or a normal use group. We sorted 42 participants (18 men and 24 women, mean age: 27.6 ± 7.2 years) into the problematic smartphone use group and 48 participants (26 men and 22 women, mean age: 30.1 ± 5.7 years) into the normal use group.

### Measures

Mindfulness was evaluated using the Five Fact Mindfulness Questionnaire–Short Form (FFMQ-SF) [56] and the Korean version of the Mindful Attention Awareness Scale (MAAS) [57]. The MAAS consists of 15 items on a Likert scale from 1 to 6 and focuses on the awareness aspects of mindfulness. The FFMQ-SF consists of 24 items on a Likert scale ranging from 1 to 5 points. The FFMQ-SF consists of five subscales: observing (4 items), describing (5 items), acting with awareness (5 items), non-judgement (5 items) and non-reactivity (5 items). For the MAAS and FFMQ-SF, higher scores indicate higher levels of mindfulness. The MAAS has been widely used as a tool to measure trait mindfulness in several studies targeting problematic smartphone use [21, 32]. The FFMQ-SF has the advantage of enriching the clinical interpretation of results because it can assess the subfactors of mindfulness in a multifaceted manner.

Impulsivity was assessed using the Korean version of the Barratt Impulsiveness Scale (BIS) [43], which consists of three subscales: attentional impulsivity (11 items), motor impulsivity (10 items) and non-planning impulsivity (9 items). The Korean version of the BIS consists of 30 items on a Likert scale ranging from 1 to 4 points. The higher scores of BIS indicate higher levels of impulsivity. The participants’ selective, sustained and divided attention was assessed through a computerized comprehensive attention test (CAT) [58], validated for adults by Huh et al. [59], which evaluates levels of various attention abilities and identifies attention deficits. When assessing selective attention, the pictures appear in the centre of a computer screen and the participants must press the button as quickly as they see the given targets (e.g., shape ‘O’). For the sustained attention test, participants press the button whenever the pictures appear, but not when the picture shape is ‘X’. Finally, in the divided attention test, a picture and sound are presented simultaneously. The participants must remember the previous picture and sound and press the button if the current picture or sound is the same as the one before. We used two indicators for each test: commission errors, the number of incorrect responses to stimuli other than the target, and omission errors, the number of missed responses to the target stimuli. Commission errors reflect impulsivity, while omission errors reflect inattention [60].

Problematic smartphone use is usually considered as one of behavioural addictions, and it has been reported to be highly correlated with substance addictions such as alcohol and nicotine use disorders [61, 62]. Therefore, we investigated whether the subjects smoked and how much they smoked, and used the Korean version of the Alcohol Use Disorders Identification Test (AUDIT) [63] to evaluate alcohol use problems. We also assessed the participants’ levels of Internet addiction using the Korean version of the Internet Addiction Test (IAT) [64].

### Statistical analysis

We used the Statistical Package for the Social Sciences (SPSS) version 22.0 (SPSS Inc., Chicago, IL, USA) and a significance level of *p* < 0.05 (two-tailed) for all statistical analyses. Independent *t*-tests and χ^2^ tests were performed to compare sociodemographic variables, clinical variables and the attention abilities of the two groups. In the group comparison of the BIS and FFMQ-SF subscales, we applied the Bonferroni correction for multiple comparisons.

Before the mediation analysis, we conducted a correlation analysis between SAPS, BIS, CAT and FFMQ-SF for all subjects. We then used model 4 of PROCESS Macro version 4.2 [65] to explore the mediating effect of the BIS subscales in the relationship between the subscales of FFMQ-SF and SAPS. The mediation model was tested at a 95% confidence interval (CI) with 5,000 bootstrap samples. We performed several mediation analyses in which each FFMQ-SF subscale has an impact on SAPS through BIS subscales; for these analyses, the other subscales of FFMQ-SF were used as covariates [65].

## Results

### Sociodemographic and clinical differences between groups

The problematic smartphone use group and the normal use group did not show differences in age, gender or FSIQ (Table 1), but the SAPS score in the normal use group was significantly lower than that in the problematic smartphone usage group (*t* = −11.631, *p* < 0.001). The groups showed no significant differences in smoking or drinking habits. The problematic smartphone use group showed significantly higher BDI, BAI and IAT scores than the normal use group (BDI: *t* = 4.572, *p* < 0.001; BAI: *t* = 4.305, *p* < 0.001; IAT: *t* = 9.463, *p* < 0.001), but the AUDIT scores were not significantly different.

**Table 1:** Comparison of sociodemographic and clinical valuables between groups

		Normal use group (*n* = 48)	Problematic smartphone use group (*n* = 42)	t/x^2^	p
Age		30.1 ± 5.7	27.6 ± 7.2	1.811	0.074
Gender (n, %)	Male	26 (54.2)	18 (42.9)	1.147	0.284
	Female	22 (45.8)	24 (57.1)		
FSIQ		111.6 ± 12.2	110.7 ± 10.2	0.390	0.698
SAPS		29.8 ± 5.8	43.7 ± 5.5	−11.631	0.000
Smoking (n, %)		7 (14.6)	4 (9.5)	0.534	0.465
Years smoking		9.9 ± 7.3	6.8 ± 5.1	0.751	0.472
Cigarettes per day		8.2 ± 4.4	6.8 ± 4.9	0.481	0.642
Drinking (n, %)		34 (70.8)	30 (71.4)	0.004	0.950
Prevalence of drinking		1.0 ± 0.8	0.9 ± 0.7	0.511	0.611
Alcohol units per drinking		1.2 ± 1.2	0.8 ± 0.6	1.674	0.100
BDI		4.3 ± 3.8	8.1 ± 4.0	4.572	0.000
BAI		3.5 ± 4.6	8.4 ± 5.9	4.305	0.000
IAT		26.3 ± 10.9	51.3 ± 14.1	9.463	0.000
AUDIT		6.3 ± 5.6	6.0 ± 6.2	−0.220	0.826

*Note* AUDIT = Alcohol Use Disorders Identification Test; BAI = Beck Anxiety Inventory; BDI = Beck Depression Inventory; FSIQ = Full-Scale Intelligence Quotient; SAPS = Smartphone Addiction Proneness Scale

### Differences in impulsivity, mindfulness and attention abilities between groups

The problematic smartphone use group appeared to have significantly more impulsivity than the normal use group (*t* = 4.349, *p* < 0.001; Table 2), showing higher scores on all BIS subscales (attentional impulsivity: *t* = 3.707, *p* < 0.001; motor impulsivity: *t* = 2.558, *p* = 0.012; non-planning impulsivity: *t* = 3.493, *p* = 0.001).

**Table 2:** Comparison of impulsivity and mindfulness between groups

	Normal use group (*n* = 48)	Problematic smartphone use group (*n* = 42)	t	p
BIS	55.6 ± 9.3	64.4 ± 9.9	4.349	0.000
Attentional	14.9 ± 3.2	18.0 ± 4.6	3.707	0.000
Motor	23.7 ± 5.2	26.3 ± 4.6	2.558	0.012
Non-planning	17.0 ± 3.6	20.0 ± 4.5	3.493	0.001
FFMQ-SF	87.8 ± 9.7	78.8 ± 9.9	−4.305	0.000
Observing	13.3 ± 2.7	13.1 ± 3.4	−0.203	0.840
Describing	19.7 ± 2.9	17.8 ± 3.8	−2.637	0.010
Acting with awareness	20.2 ± 2.5	17.5 ± 3.8	−3.952	0.000
Non-judgement	17.0 ± 4.1	14.5 ± 4.5	−2.671	0.009
Non-reactivity	17.6 ± 3.1	15.8 ± 3.8	−2.383	0.019
MAAS	74.1 ± 6.9	64.5 ± 11.2	−4.826	0.000

*Note* BIS = Barratt Impulsiveness Scale; FFMQ-SF = Five Facet Mindfulness Questionnaire–Short Form; MAAS = Mindful Attention Awareness Scale

The problematic smartphone use group also showed lower scores on both measures of mindfulness (FFMQ-SF: *t* = -4.305, *p* < 0.001; MAAS: *t* = 4.826, *p* < 0.001). After the Bonferroni correction (*p* < 0.05/5), the problematic smartphone use group showed significantly lower scores on the acting with awareness and non-judgement subscales of FFMQ-SF (acting with awareness: *t* = −3.952, *p* < 0.001; non-judgement: *t* = −2.671, *p* = 0.009).

There was no significant difference in CAT indexes between the two groups (Table 3). Only the difference in selective attention omission errors was close to being statistically significant (*t* = 1.910, *p* = 0.063).

**Table 3:** Group differences in the indexes of comprehensive attention test

	Normal use group (*n* = 48)	Problematic smartphone use group (*n* = 42)	t	p
**Selective attention**				
Omission error	0.0 ± 0.1	0.3 ± 1.1	1.910	0.063
Commission error	0.5 ± 0.9	0.7 ± 1.3	0.796	0.428
**Sustained attention**				
Omission error	1.3 ± 2.7	0.7 ± 1.2	−1.234	0.222
Commission error	4.7 ± 4.8	5.8 ± 6.3	0.961	0.339
**Divided attention**				
Omission error	3.0 ± 3.1	3.9 ± 3.6	1.363	0.176
Commission error	2.7 ± 1.4	2.8 ± 1.6	0.105	0.916

### Correlation between clinical variables

The SAPS and BIS scores, including the subscales, were all positively correlated (total BIS: *r* = 0.477, *p* < 0.001; attentional impulsivity: *r* = 0.453, *p* < 0.001; motor impulsivity: *r* = 0.322, *p* = 0.002; non-planning impulsivity: *r* = 0.340, *p* < 0.001; Table 4). The correlations between the SAPS and both mindfulness scales were significantly negative (FFMQ-SF: *r* = −0.428, *p* < 0.001; MAAS: *r* = −0.481, *p* < 0.001). After the Bonferroni correction (*p* < 0.05/5), acting with awareness and non-reactivity subscales of FFMQ-SF showed significant correlations with the SAPS (acting with awareness: *r* = −0.448, *p* < 0.001; non-reactivity: *r* = −0.329, *p* = 0.002). Furthermore, the total BIS and mindfulness scores were all negatively correlated (FFMQ-SF: *r* = −0.498, *p* < 0.001; MAAS: *r* = −0.626, *p* < 0.001).

**Table 4:** Correlation matrix

	1	2	2-1	2-2	2-3	3	3-1	3-2	3-3	3-4	3-5	4	5-1	5-2	5-3	5-4	5-5	5-6	6	7
**1. SAPS**	1																			
**2. BIS**	0.477***	1																		
2-1. Attentional	0.453***	0.712***	1																	
2-2. Motor	0.322**	0.790***	0.279**	1																
2-3. Non-planning	0.340**	0.810***	0.430***	0.471***	1															
**3. FFMQ-SF**	−0.428***	−0.498***	0.347**	−0.469***	−0.322***	1														
3-1. Observing	0.006	−0.137	0.129	−0.341**	−0.056	0.370***	1													
3-2. Describing	−0.248*	−0.387***	−0.133	−0.367***	−0.379***	0.703***	0.160	1												
3-3. Acting with awareness	−0.448***	−0.571***	−0.489***	−0.315**	−0.544***	0.585***	−0.086	0.484***	1											
3-4. Non-judgement	−0.233*	−0.081	−0.236*	−0.062	0.108	0.603***	0.015	0.144	0.123	1										
3-5. Non-reactivity	− 0.329**	− 0.355**	− 0.258*	− 0.383***	− 0.160	0.699***	0.169	0.361***	0.243*	0.302**	1									
**4. MAAS**	− 0.481***	− 0.626***	− 0.604***	− 0.329**	0.548***	0.573***	− 0.123	0.430***	0.863***	0.220*	0.305**	1								
**5. CAT**																				
5-1. Selective attention - Omission error	0.152	0.021	0.030	0.010	0.009	0.079	0.026	0.041	− 0.001	− 0.226*	− 0.019	− 0.055	1							
5-2. Selective attention - Commission error	0.031	0.080	0.106	0.044	0.039	0.074	0.102	0.047	− 0.052	0.005	0.035	− 0.012	0.276**	1						
5-3. Sustained attention - Omission error	− 0.174	0.084	0.154	0.033	0.015	0.010	0.063	− 0.045	− 0.088	0.042	0.051	− 0.041	− 0.056	0.204	1					
5-4. Sustained attention - Commission error	0.050	0.184	0.201	0.058	0.185	− 0.065	0.093	− 0.042	− 0.110	− 0.032	− 0.085	− 0.178	0.013	0.594***	0.147	1				
5-5. Divided attention - Omission error	0.178	− 0.013	0.090	− 0.087	− 0.017	0.056	0.095	0.091	− 0.048	0.091	− 0.066	− 0.031	0.107	− 0.126	− 0.025	0.022	1			
5-6. Divided attention - Commission error	0.038	− 0.018	0.042	− 0.003	− 0.081	0.070	− 0.019	0.199	0.091	0.044	− 0.106	0.093	0.061	0.241*	0.018	0.387***	0.198	1		
**6. BDI**	0.423***	0.397***	0.362***	0.247*	0.310**	−0.546***	0.019	−0.307**	−0.391***	−0.438***	−0.440***	−0.484***	0.211*	0.037	0.211*	0.037	0.079	−0.027	1	
**7. BAI**	0.480***	0.467***	0.585***	0.285**	0.239*	−0.442***	0.266*	−0.212*	−0.475***	−0.373**	−0.429***	−0.564***	0.034	0.165	0.034	0.165	0.026	0.118	0.515***	1

*Note* CAT = Comprehensive Attention Test; SAPS = Smartphone Addiction Proneness Scale; BIS = Barratt Impulsiveness Scale; FFMQ = Five Facet Mindfulness Questionnaire; MAAS = Mindful Attention Awareness Scale; BDI = Beck Depression Inventory; BAI = Beck Anxiety Inventory

The CAT indexed had no significant correlation with SAPS, FFMQ-SF, MAAS, and BIS scores. The BDI showed significant correlation with SAPS, FFMQ-SF, MAAS and BIS scores (SAPS: *r* = 0.423, *p* < 0.001; FFMQ-SF: *r* = −0.546, *p* < 0.001; MAAS: *r* = −0.484, *p* < 0.001; BIS: *r* = 0.397, *p* < 0.001). The BAI showed significant correlation with SAPS, FFMQ-SF, MAAS and BIS scores (SAPS: *r* = 0.480, *p* < 0.001; FFMQ-SF: *r* = -0.442, *p* < 0.001; MAAS: *r* = −0.564, *p* < 0.001; BIS: *r* = 0.467, *p* < 0.001).

### The mediating effect of impulsivity between mindfulness and smartphone addiction

The mediation analysis identified only the acting with awareness subscale of FFMQ-SF as a significant predictor of SAPS scores (total effect β = 1.039, SE = 0.283, 95% CI [−1.604; 0.475]; Table 5). We found a significant indirect effect of the impact of acting with awareness on SAPS scores through attentional impulsivity (β = −0.287, SE = 0.150, 95% CI [−0.609; 0.017]); however, the direct effect of acting with awareness on SAPS scores was not significant. Taken together, attentional impulsivity completely mediated the relationship between acting with awareness and the SAPS score.

**Table 5:** The mediating effect of BIS subscales between FFMQ-SF subscales and SAPS in total subjects (*n* = 90)

Independent variable	Dependent variable	Total effect	Direct effect	Mediators	Indirect effect	Confidence interval	
						Lower bound	Upper bound
Observing	SAPS	0.006 (*p* = 0.983)	0.063 (*p* **=** 0.834)	Attentional impulsivity	0.056	− 0.081	0.264
				Motor impulsivity	− 0.098	− 0.333	0.134
				Non-planning impulsivity	− 0.015	− 0.123	0.097
Describing		0.101 (*p* = 0.732)	0.061 (*p* **=** 0.840)	Attentional impulsivity	0.103	− 0.041	0.276
				Motor impulsivity	− 0.037	− 0.230	0.054
				Non-planning impulsivity	− 0.026	− 0.166	0.085
Acting with awareness		-1.039 (*p* = 0.000)	− 0.598 (*p* **=** 0.073)	Attentional impulsivity	− 0.287	− 0.609	− 0.017
				Motor impulsivity	− 0.065	− 0.250	0.093
				Non-planning impulsivity	− 0.089	− 0.440	0.230
Non-judgement		− 0.255 (*p* = 0.203)	− 0.232 (*p* **=** 0.263)	Attentional impulsivity	− 0.066	− 0.203	0.022
				Motor impulsivity	0.015	− 0.048	0.108
				Non-planning impulsivity	0.028	− 0.074	0.150
Non-reactivity		− 0.524 (*p* = 0.051)	− 0.355 (*p* **=** 0.193)	Attentional impulsivity	− 0.092	− 0.307	0.020
				Motor impulsivity	− 0.070	− 0.280	0.083
				Non-planning impulsivity	− 0.006	− 0.105	0.072

*Note* BIS = Barratt Impulsiveness Scale; FFMQ-SF = Five Facet Mindfulness Questionnaire–Short Form; SAPS = Smartphone Addiction Proneness Scale

## Discussion

In this study, we compared several psychological characteristics of problematic smartphone users and normal users. The problematic smartphone use group had higher scores for negative emotions, such as depression and anxiety, than the normal use group. These findings are consistent with previous studies that reported a close association between problematic smartphone usage and emotional problems [66, 67]. The comparison of our subjects also suggested that problematic smartphone users had lower levels of mindfulness than the normal use group, consistent with previous studies showing low levels of mindfulness in addictive diseases [68]. In this study, problematic smartphone users showed significant differences from the normal use group in awareness and non-judgement among several sub-factors of mindfulness. In the context of mindfulness, awareness is the ability to notice what thoughts, feelings and sensations are currently going on inside oneself [33]. Loss of control over addictive behaviour is closely related to decreased awareness of interoceptive signals derived from addictive behaviour [69]. Our results suggest that a tendency to act without sufficient awareness may lead to a loss of control over excessive smartphone use. On the other hand, a non-judgemental attitude has been negatively correlated with affective symptoms, such as depression and anxiety [70]. Thus, we speculate that low levels of non-judgement in problematic smartphone users may be associated with their high tendency toward emotional difficulties.

We also compared trait impulsivity assessed through self-reports and attentional capacity assessed through computerized attention tests between problematic and normal smartphone users. Problematic smartphone users reported a higher level of trait impulsivity than normal users, which is consistent with previous studies of impulsiveness in problematic smartphone users [61]. On the other hand, the CAT results showed no difference between the groups’ omission and commission errors of selective, sustained and divided attention. A previous study reported that the adverse effects of smartphone use on general attention capacity are not remarkable in the general population [71]. A recent study also reported no significant impairment in overall cognitive function as assessed through behavioral tasks in problematic smartphone use [72]. Consistent with these previous studies, our current findings suggest that impairments in general attentional function are not evident for problematic smartphone use. Previous studies that reported attentional control deficit in problematic smartphone use mainly focused on and evaluated attentional control in the presence of interfering stimuli [36, 37]. In the current study, we did not conduct the Flanker test, which measures one’s ability to respond to target stimuli while ignoring interfering stimuli. Thus, attentional control in the presence of distractions in problematic smartphone use requires further investigation.

Although commission errors, which are inappropriate responses to non-goals, have been recognized as indicators of impulsivity in cognitive tasks [73], our findings found no correlation between these CAT indicators and BIS scores. However, previous studies measuring impulsivity often report conflicting results between self-report and cognitive tasks [74–76]. Impulsivity can be conceptualized and measured in many ways, and different cognitive tasks and self-report questionnaires measure different aspects of impulsivity. For example, three-dimensional constructs of impulsivity, including impulsive choice, behaviour and personality traits, have been proposed [77]. A behavioural cognitive task usually measures impulsive behaviour, whereas self-reports measure impulsive personality traits. Therefore, our current findings suggest that although impulsive behaviour is not prominent in problematic smartphone users, impulsive personality traits are. These results are consistent with prior evidence that impulsive personality traits are linked to vulnerability to addictive disorders [78]. Another thing to consider is that the inhibitory control deficits in behavioural addictions are stimulus-specific inhibitory control deficits rather than general inhibitory control deficits [47]. A previous study reported that commission errors were more frequent in problematic smartphone users only when exposed to the mobile phone-related stimulus [79]. The CAT in this study was not conducted within the context in which smartphone-related stimuli were presented. Therefore, the results of this study suggest that impulsive behaviours related to general inhibitory control were not prominent in problematic smartphone use, and should be interpreted taking into account that no smartphone-related stimuli were presented.

In this study, we investigated the relationship between mindfulness, impulsivity and addictive smartphone use. Prior evidences have shown that impulsivity is associated with mindfulness [80, 81] and addictive behaviour [82, 83]. Consistent with previous studies, BIS scores showed significant correlations with FFMQ-SF, MAAS and SAPS scores in this study’s correlation analysis. We further explored whether impulsivity plays a mediating role between mindfulness and addictive smartphone use and found that the acting with awareness facet of mindfulness affects addictive smartphone use through attentional impulsivity. This attentional impulsivity is one of the aspects of trait impulsivity assessed through the BIS, and refers to impulsiveness related to attentional control performance [84]. Since attentional control is a major domain of executive function [85], attentional impulsivity may be considered to represent impulsiveness during executive functioning. Consistent with this speculation, previous research on trait impulsivity has suggested that attentional impulsivity is correlated with cognitive processes, such as conflict monitoring and response organization, involving executive functions [45, 86, 87]. Disease models of addictive behaviour have identified diminished executive functioning as a major pathophysiological cause of loss of control over addictive behaviour [47]. Therefore, we speculate that diminished executive functioning reflected by high attentional impulsivity is linked to addictive patterns of smartphone use. Acting with awareness refers to the ability to pay attention to activities in the present moment [56, 88]. A sufficient level of awareness is a prerequisite for self-monitoring and self-regulation, which are key elements in performing higher cognitive functions, such as executive function [89]. Previous studies have suggested that mindfulness meditation and mindfulness-based interventions can enhance executive control by improving awareness [90]. Taken together, our mediation analysis results imply that diminished executive functioning, which is reflected in high attentional impulsivity, mediates the relationship between low levels of awareness and addictive smartphone use. This implication is in line with a previous study showing that mindfulness acts as a protective factor in problematic smartphone use, but high impulsivity can interfere with it [32].

The mediation analysis identified the specific role of ‘acting with awareness’ among the various facets of mindfulness, which is consistent with previous studies that identified relationships of awareness to impulsivity and problematic smartphone use [14, 29]. However, in the mediation analysis, no significant effects were found in facets of mindfulness other than awareness. In particular, ‘non-judgment’ also failed to show a significant effect in the mediation analysis model despite significant differences between groups. Previous studies on mindfulness have suggested that non-judgmental attitude is distinct from other mindfulness facets related to attention and is more closely connected to other positive psychological factors such as compassion [91]. Similarly to this, our current study suggests that ‘non-judgment’ is related to problematic smartphone use in a distinct manner from ‘acting with consciousness’. Another thing to keep in mind when interpreting this study is that only basic mediation models were explored. Relevant previous evidence suggests that there are several factors whose moderating effects should be considered. For example, one recent study found that trait mindfulness moderated the relationship between perceived stress and problematic smartphone use through experiential avoidance [92]. Another study suggested that awareness mediates between alexithymia and problematic smartphone use, while the facet of observing has a moderating effect on awareness [93]. Future studies should consider that multiple facets of mindfulness may be related to problematic smartphone use through complex pathways through moderated mediation.

The limitations of this study are as follows. First, we did not evaluate objective smartphone use patterns in the real world, only through the self-reported questionnaire scale. Given the diversity of smartphone-based online activities, collecting objective data on time consumption and behavioural patterns of online activities through smartphones would be helpful. Previous studies have suggested that self-report scales for problematic usage and objective measures of smartphone usage do not entirely correspond, and that some variables of objective measures (e.g. frequency of use and specific apps) significantly reflect problematic smartphone use [94, 95]. Considering this, future research including objective measures of smartphone usage will be able to reduce the risk of bias in self-reporting and evaluate problematic smartphone use more objectively and richly. Second, the measurement tools used to evaluate the main variables in this study were limited. Various psychometric scales to evaluate problematic smartphone use have been developed and are being used [96]. Therefore, if the subjects were cross-validated using multiple scales, the subjects’ addictive smartphone use status could have been more accurately reflected. In addition, mindfulness, impulsivity and attention are all composed of various psychometric aspects, so a comprehensive evaluation is needed. For instance, mindfulness can be defined and evaluated not only as a trait factor, as in this study, but also as a state factor during mindful attention [97]. Our study also did not include impulsive choice tasks, which can be used to evaluate a person’s tendency to choose smaller, immediate rewards over larger, delayed rewards [98]. As previously mentioned, this study also did not include the Flanker test, which evaluates the capacity to maintain attention despite a distractor. Future studies that include more diverse aspects of mindfulness, impulsivity, and attention and perform complex mediation and moderation modelling will help identify relationships between these variables more precisely. Third, although this study excluded subjects with a history of major mental illness or high levels of depression and anxiety, the influence of emotional factors could not be sufficiently excluded. The BDI and BAI scores differed across groups and were highly correlated with other psychometric variables, suggesting that differences in subjects’ depression and anxiety levels may have influenced the results. Additionally, the high correlation of the BDI and BAI with the SAPS may be related to the controversy that items in technology usage scales overlap with existing measures of psychological well-being [99]. Fourth, the number of subjects in this study was not large enough to sufficiently explore the relationship between variables. For example, structural equation modelling, which has the advantage of providing a flexible framework for analysing complex relationships between multiple variables, usually requires 200 or more subjects [100]. Fifth, this study’s cross-sectional design was limited to evaluate the causal relationship between mindfulness and problematic smartphone use. A longitudinal follow-up study is needed to explore changes in addictive smartphone use according to changes in mindfulness levels. According to previous research on longitudinal design, mindfulness and problematic smartphone use have a reciprocal relationship that influences each other [101]. These results suggest the clinical usefulness of actively managing mindfulness to improve problematic smartphone use.

In conclusion, this study evaluated the levels of mindfulness, impulsiveness and attentional capacity of problematic smartphone users and explored the relationships among the variables. Problematic smartphone users had a lower level of mindfulness than healthy users, and the difference was particularly noticeable in the acting with awareness and non-judgement facets of mindfulness. The problematic smartphone use group had higher trait impulsivity than the normal use group, but there was no difference in their performance on behavioral attention tasks. In particular, our results suggest that a low level of awareness has a significant effect on addictive patterns of smartphone use and that diminished executive control, reflected by high attentional impulsivity, mediates the relationship between awareness and addictive smartphone use. Each type of mindfulness meditation and mindfulness-based intervention has different goals as to which components of mindfulness are primarily improved. Based on the results of this study, when creating a treatment plan for problematic smartphone users, a mindfulness-based intervention that focuses on increasing awareness and improving impulsivity can be designed. Additionally, this study suggests that assessing attentional impulsivity and awareness levels along with addictive use of smartphones may be effective when determining the effectiveness of mindfulness-based interventions and tracking clinical outcomes. To summarize, the results of this study imply that mindfulness-based interventions that can promote awareness and executive control may be an effective approach to problematic smartphone use.

## Data Availability

The datasets generated during and/or analyzed during the current study are available from the corresponding author on reasonable request.
